# Reply to De Giorgi et al. Comment on “Kesić et al. Early Diagnostics of Vulvar Intraepithelial Neoplasia. *Cancers* 2022, *14*, 1822”

**DOI:** 10.3390/cancers14205088

**Published:** 2022-10-18

**Authors:** Vesna Kesić, Pedro Vieira-Baptista, Colleen K. Stockdale

**Affiliations:** 1 Medical Faculty, University of Belgrade, 11000 Belgrade, Serbia; 2 Clinic for Obstetrics and Gynecology, University Clinical Center of Serbia, 11000 Belgrade, Serbia; 3 Lower Genital Tract Unit, Centro Hospitalar de São João, 4200-319 Porto, Portugal; 4 Hospital Lusíadas Porto, 4200-319 Porto, Portugal; 5 Department of Obstetrics & Gynecology, Vulvar Vaginal Disease and Colposcopy Clinics, University Iowa Healthcare, Iowa City, IA 52242, USA

We thank you and your co-authors for the comment [1] on our article “*Early Diagnostics of Vulvar Intraepithelial Neoplasia*” published in *Cancers* 2022, *14*, 1822 [2]. It is a pleasure to see that our article drew attention, particularly from the side of dermatologists. Vulvar pathology, indeed, comprises the spectrum of different conditions and diseases that require a multidisciplinary approach involving gynecologists, dermatologists, and pathologists.

Dermoscopy is without doubt an extremely useful diagnostic tool in the evaluation of pigmented lesions on vulvar surface, particularly in suspicion of melanocitic lesions. However, dermoscopy is not available to non-dermatologists. In gynecological practice, whenever the suspicion of melanoma is raised, dermatologists are necessarily involved into a team. Additionally, most of the patients with dermatoses are sent to dermatologists who decide if dermoscopy or confocal microscopy would be appropriate, particularly for non-melanocitic lesions. 

Our article is focused to squamous vulvar intraepithelial neoplasia (VIN). Dermoscopy does help differentiating infective and inflammatory conditions of the vulva and aids in avoiding unnecessary biopsies. However, VIN has a specific, most often easily recognizable vulvoscopic appearance, and since its capacity for progression to cancer is of utmost importance, biopsy or local excision are essential. Pigmented VIN lesions are not difficult to differ from melanoma, and thus dermoscopy and confocal microscopy are not routinely used for the diagnosis of VIN. They are addressed from time to time, but not enough evidence was ever shown that dermoscopy and confocal microscopy should lead to the recommendation of using it in gynecological clinical practice.

Regardless, your comment is greatly appreciated. It is an invaluable addition to Barisani’s [3] and Vaccari’s [4] articles, which together with a few other articles published by dermatologists, particularly the one prepared by Kavita and Deeksha [5], emphasize the need for closer cooperation between gynecologists and dermatologists in management of vulvar diseases.
